# Supplementary material for: The secondary somatosensory cortex gates mechanical and heat sensitivity
Source: Nat Commun. 2024 Feb 12;15:1289. doi: 10.1038/s41467-024-45729-7 (PMC10861531; doi:10.1038/s41467-024-45729-7)
Supplement: Supplementary file 1 — Supplementary Information [file 41467_2024_45729_MOESM1_ESM.pdf]

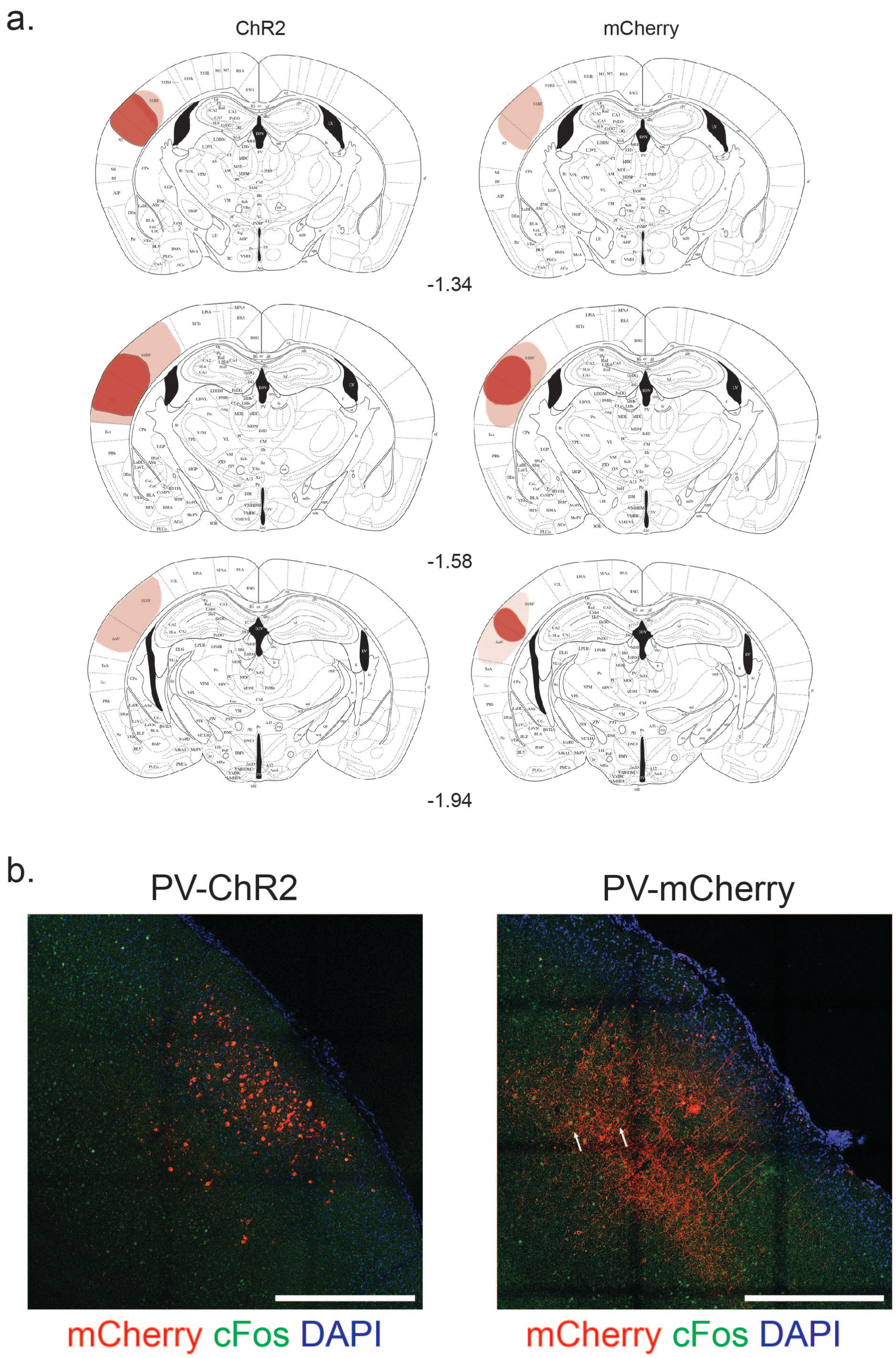

Supplementary Figure 1: Channelrhodopsin and mCherry viral injection coverage in PV-Cre mice
a. Averaged spread of virus injection in mice across different anterior-posterior ranges. Darker red indicates more intense expression whereas lighter red is lower expression. For all groups n=7. b. Representative images of cFos staining following blue light stimulation in PV-ChR2 and PV-mCherry animals. See arrows for example cFos positive neurons. Scale bar: 500μM.

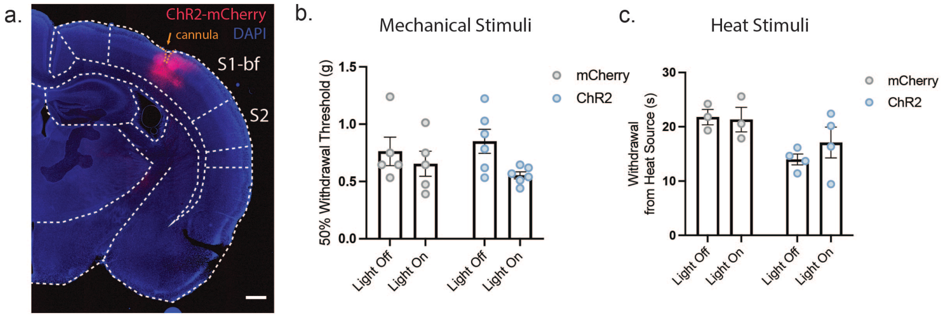

Supplementary Figure 2: Inhibition of the adjacent S1-barrel cortex does not produce mechanical or thermal sensitivity in the hindpaw.
(a): Representative image of ChR2 expression and fiber placement in the S1-barrel cortex of PV-Cre mice. Scale bar – 500 µm. (b). Light stimulation of mice with ChR2 expression in S1-barrel cortex display no tactile hypersensitivity by von Frey. n=5 mCherry, n=6 ChR2. Two Way ANOVA with Bonferroni's. mCherry light on vs. off $p=0.9129$. ChR2 light on vs. off $p=0.0690$. (c). Light stimulation of mice with ChR2 expression in S1-barrel cortex display no heat hypersensitivity by Hargreaves'. n=3 mCherry, n=4 ChR2. Two Way ANOVA with Sidak's. mCherry light on vs. off $p>0.9999$. ChR2 light on vs. off $p=0.8653$.

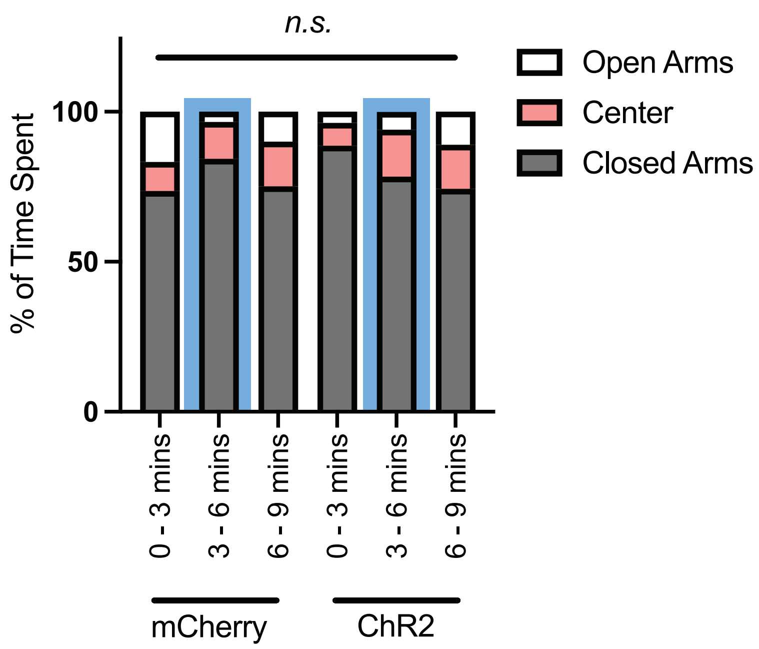

Supplementary Figure 3: S2 inhibition does not induce anxiolytic behavior.
Elevated plus maze total results examining the % of time spent in the closed, center, and open arms. A baseline period was recorded from 0-3 mins, blue light illumination (S2 inhibition) occurred between 3-6 mins, and a recovery period from 6-9 mins. No significant differences observed between mCherry control (n=7) and ChR2 mice (n=6). Two Way ANOVA with Sidak's.

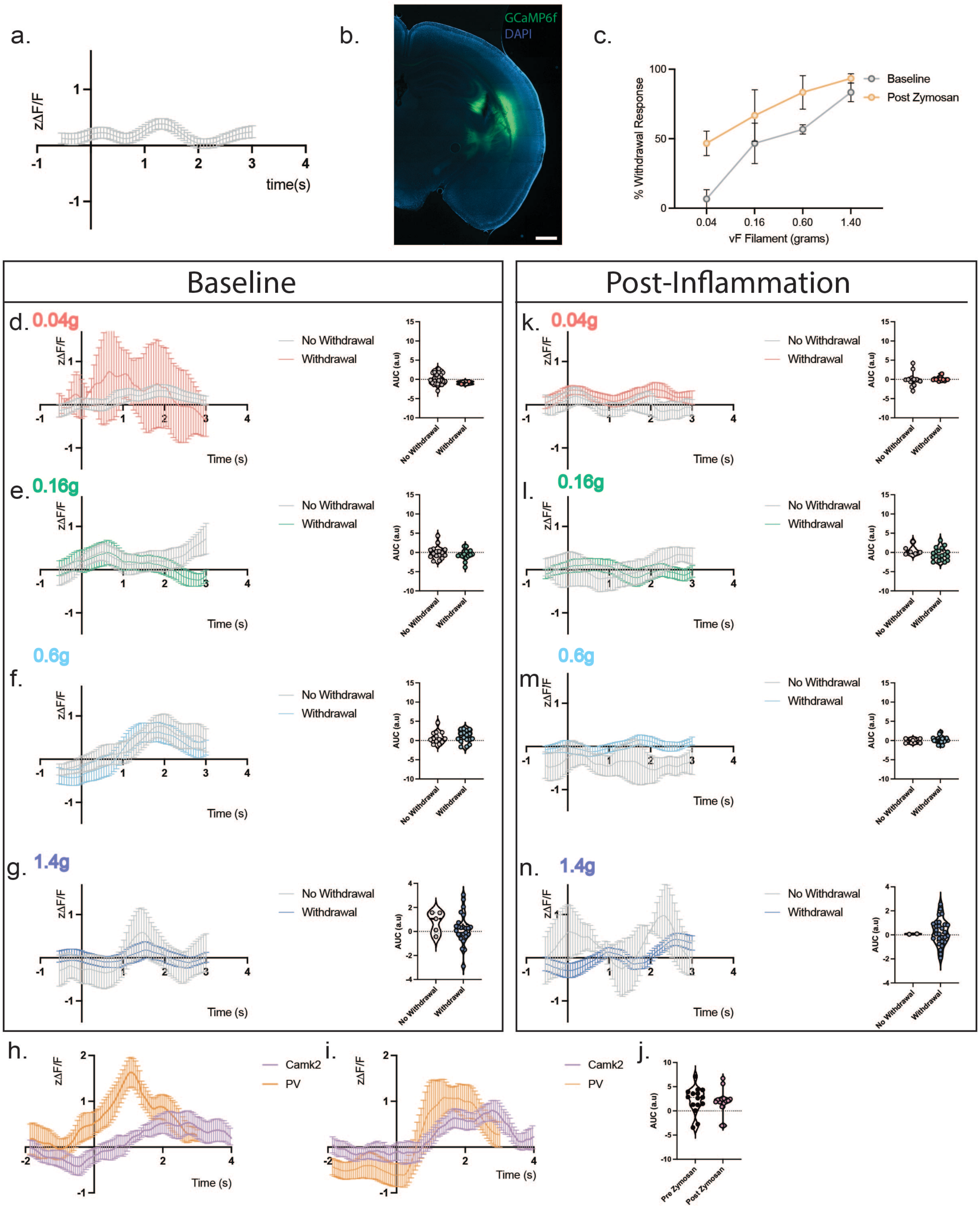
a.
zΔF/F
time(s)
b.
GCaMP6f
DAPI
c.
% Withdrawal Response
vF Filament (grams)
Baseline
Post Zymosan
Baseline
Post-Inflammation
d. 0.04g
e. 0.16g
f. 0.6g
g. 1.4g
k. 0.04g
l. 0.16g
m. 0.6g
n. 1.4g
No Withdrawal
Withdrawal
Time (s)
AUC (a.u)
h.
i.
j.
Camk2
PV
Pre Zymosan
Post Zymosan

Supplementary Figure 4: Calcium dynamics of CaMKII positive excitatory neurons in S2 at baseline and following hindpaw inflammation.

(a): Averaged Z-scored delta F/F of non-stimulus evoked hindpaw withdrawal. Time 0 is aligned to the beginning of the hindpaw withdrawal. (b): Example of GCaMP6f expression in CaMKII neurons within the S2 region. Scale bar = 500µm. (c): Percent withdrawal to differentially weighted mechanical von Frey stimulation of the hindpaw in fiber implanted animals both before and after inflammatory induction. (d,e,f,g): Left: Calcium responses of CaMKII neurons in S2 to a 0.04, 0.16, 0.6 and 1.4g mechanical stimulus plotted by Z-scored delta F/F following a single stimulation of the hindpaw at time 0. Right: Area under curve analysis of calcium transients produced in d, e, f, g. Unpaired t-test. (h): Baseline calcium responses in both CaMKII and PV neurons (data transposed from Figure 2) in S2 plotted by Z-scored delta F/F to a heat ramp stimulus. Time 0 is time of paw withdrawal from the heat source. (i): Calcium responses in both CaMKII and PV neurons (data transposed from Figure 2) in S2 plotted by Z-scored delta F/F to a heat ramp stimulus after zymosan induced inflammation. Time 0 is time of paw withdrawal from the heat source. (j): Area under curve analysis of the calcium transients produced during heat stimulus trials depicted in i and j. (l, m, n, o): Left: Calcium responses of PV neurons in S2 plotted by Z-scored delta F/F following a single mechanical stimulation of the hindpaw at time 0, 4 hours post zymosan injection. Force of stimulation (between 0.04g-1.4g) displayed in the graphs. Right: Area under curve analysis of calcium transients produced in l, m, n, o. Unpaired t-test. For all experiments, n=3 animals, 5-10 sensory trials per mouse, see Methods. Data presented as mean ± SEM. *p=<0.05, **p=<0.005, ***p=<0.0005.

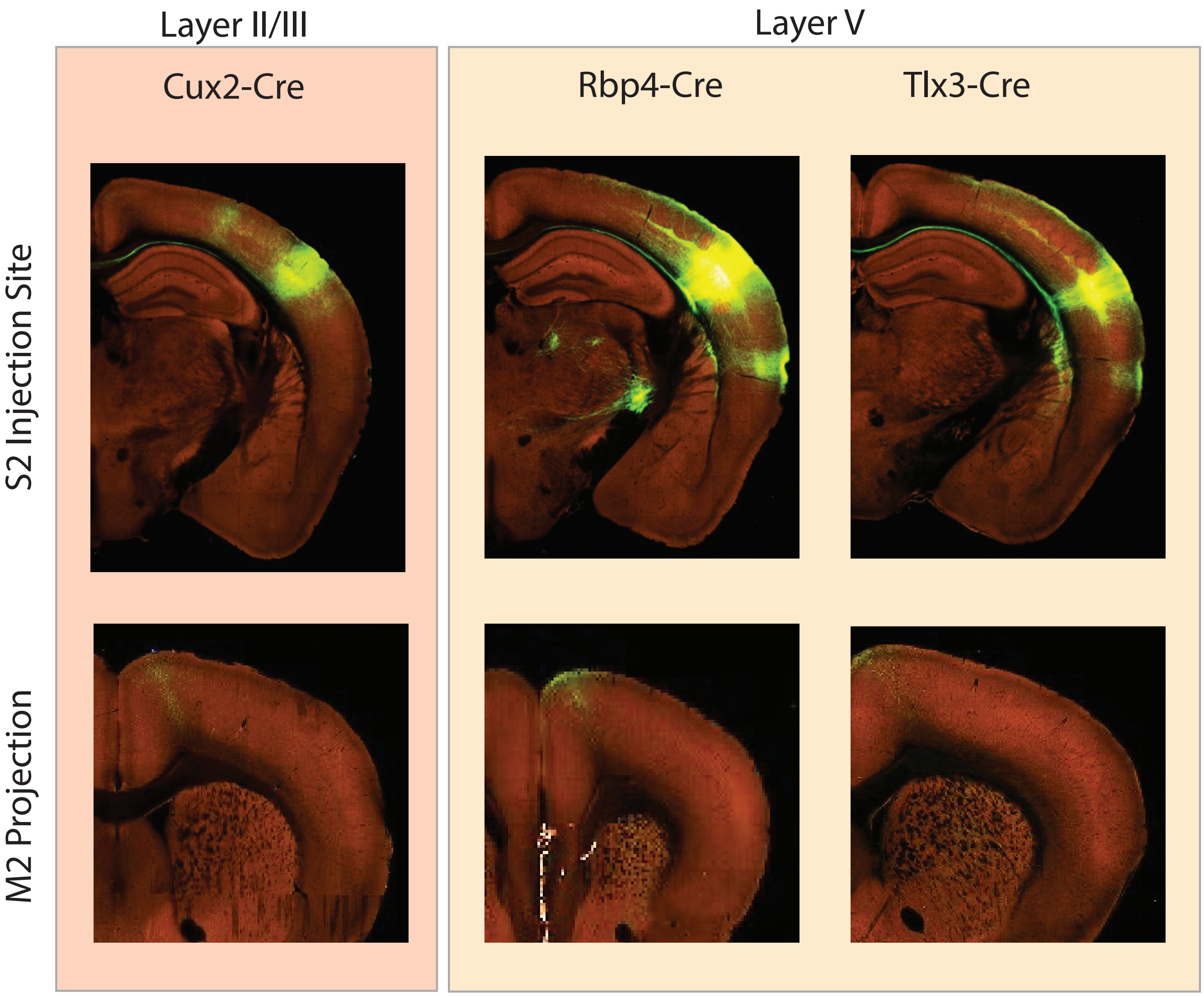

Supplementary Figure 5: Layer specific contributions of S2 projections to M2.
Representative images from the Allen Brain Connectome Atlas (https://connectivity.brain-map.org)[42,54]. Cre-dependent AAV-GFP was injected into S2 of various cre lines (*Cux2* for layer II/III and *Rbp4* or *Tlx3* for layer V) and projections to M2 are clearly identified with largest portion occurring from Rbp4-Cre animals.

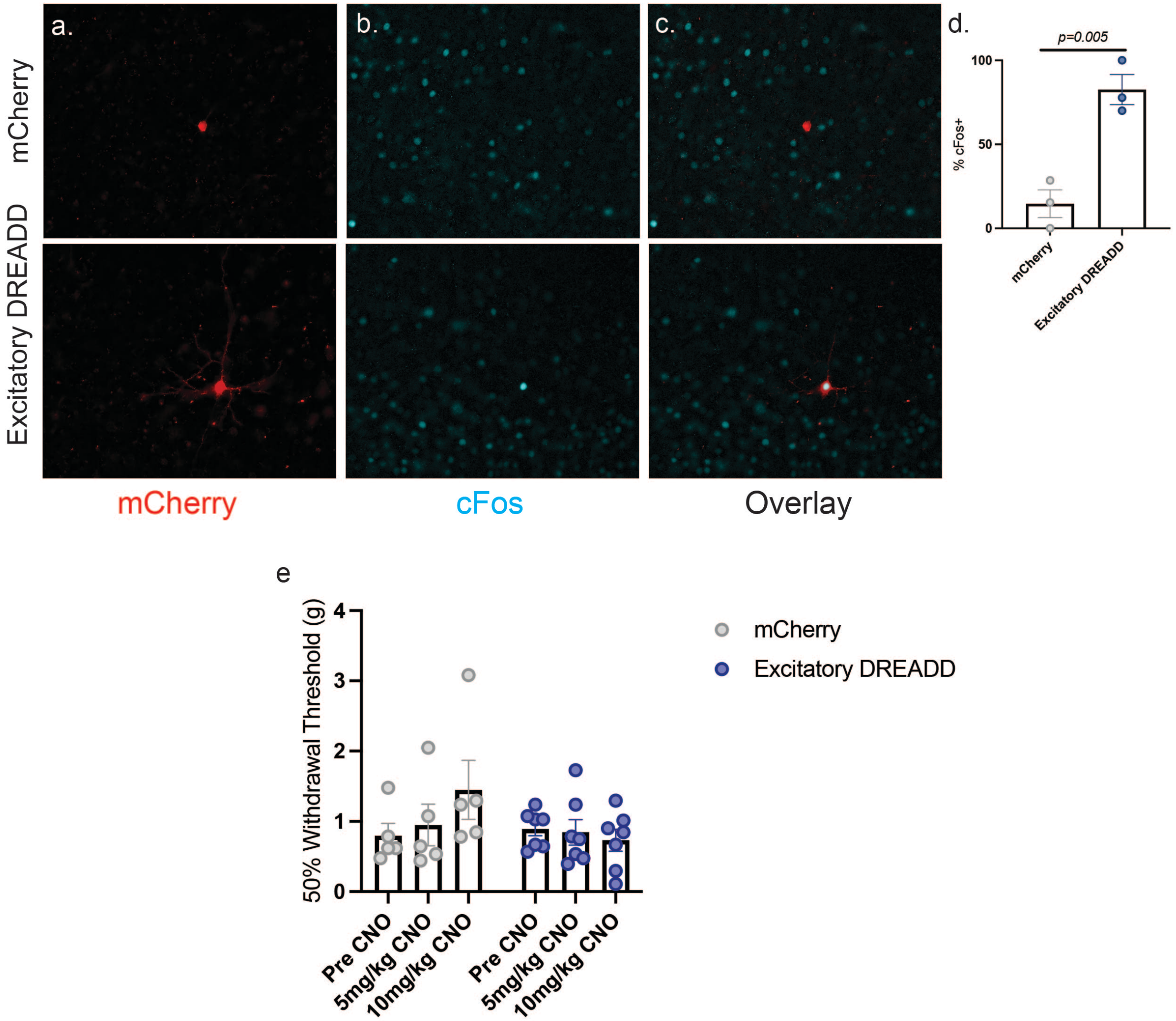

Supplementary Figure 6: CNO administration increases cFos expression in excitatory DREADD labelled S2-to-M2 neurons but does not influence somatosensory behavior.
(a): Representative images showing S2-to-M2 virally labeled neurons with AAV-hSyn-DIO-mCherry or AAV-hSyn-DIO-Hm3q-mCherry (excitatory DREADD). (b): cFos staining in the same plane as in A. (c): Overlay between mCherry labeled neurons and cFos. (d): Quantification of % of mCherry-positive neurons that colabel with cFos. (n=3 animals per group, unpaired t-test). (e): Effect of different doses of clozapine-n-oxide (CNO) on mCherry control and S2-to-M2 excitatory DREADD animals (Two Way ANOVA with Tukey's, n.s.). Data presented as mean ± SEM.

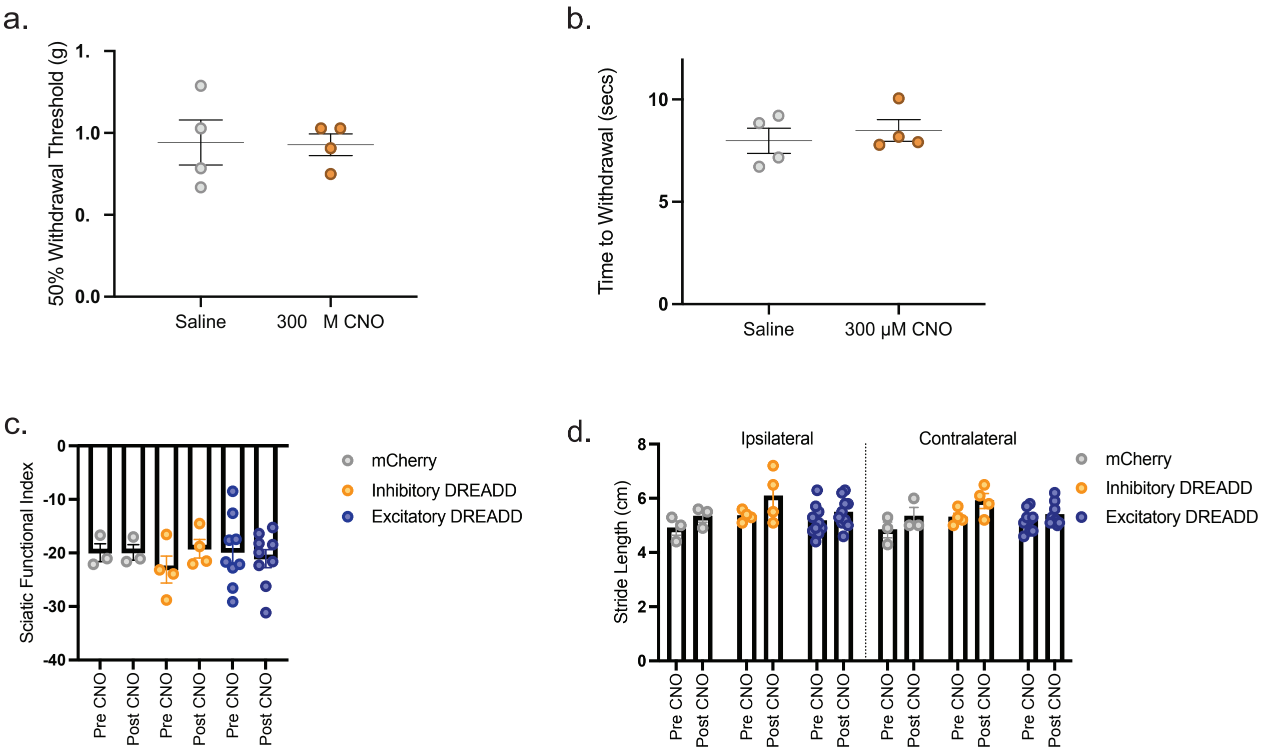

Supplementary Figure 7: Local Injection of CNO into M2 does not alter sensory behavior in wildtype mice and S2 inhibition does not alter gross motor behavior.

(a): Local injection of 300 nanoliters of 300µM clozapine-n-oxide (CNO) into the M2 region into control animals without viral expression via cannula does not alter mechanical sensitivity. n=4 for all groups.

(b): Local injection of 300 nanoliters of 300µM clozapine-n-oxide (CNO) into the M2 region into control animals without viral expression via cannula does not alter heat sensitivity. n=4 for all groups.

(c): Hindpaw placement during locomotion (sciatic functional index) was unaffected in S2-to-M2 chemogenetically inhibited or excited mice. mCherry n=4, Inhibitory DREADD n=5, Excitatory DREADD n=9. Two Way ANOVA.

(d): Stride length was unaffected in S2-to-M2 chemogenetically inhibited or excited mice. mCherry n=4, Inhibitory DREADD n=5, Excitatory DREADD n=9. Two Way ANOVA.

All data presented as mean ± SEM.
