# Supplementary material for: The secondary somatosensory cortex gates mechanical and heat sensitivity
Source: Nat Commun. 2024 Feb 12;15:1289. doi: 10.1038/s41467-024-45729-7 (PMC10861531; doi:10.1038/s41467-024-45729-7)
Supplement: Supplementary file 3 — Reporting Summary [file 41467_2024_45729_MOESM3_ESM.pdf]

nature portfolio

Corresponding author(s): Clifford J. Woolf

Last updated by author(s): Jan 24, 2024

Reporting Summary

Nature Portfolio wishes to improve the reproducibility of the work that we publish. This form provides structure for consistency and transparency in reporting. For further information on Nature Portfolio policies, see our Editorial Policies and the Editorial Policy Checklist.

Statistics

For all statistical analyses, confirm that the following items are present in the figure legend, table legend, main text, or Methods section.

n/a	Confirmed	
☐	☒	The exact sample size (*n*) for each experimental group/condition, given as a discrete number and unit of measurement
☐	☒	A statement on whether measurements were taken from distinct samples or whether the same sample was measured repeatedly
☐	☒	The statistical test(s) used AND whether they are one- or two-sided<br>*Only common tests should be described solely by name; describe more complex techniques in the Methods section.*
☐	☒	A description of all covariates tested
☐	☒	A description of any assumptions or corrections, such as tests of normality and adjustment for multiple comparisons
☐	☒	A full description of the statistical parameters including central tendency (e.g. means) or other basic estimates (e.g. regression coefficient) AND variation (e.g. standard deviation) or associated estimates of uncertainty (e.g. confidence intervals)
☐	☒	For null hypothesis testing, the test statistic (e.g. *F*, *t*, *r*) with confidence intervals, effect sizes, degrees of freedom and *P* value noted<br>*Give P values as exact values whenever suitable.*
☒	☐	For Bayesian analysis, information on the choice of priors and Markov chain Monte Carlo settings
☒	☐	For hierarchical and complex designs, identification of the appropriate level for tests and full reporting of outcomes
☒	☐	Estimates of effect sizes (e.g. Cohen's *d*, Pearson's *r*), indicating how they were calculated

Our web collection on statistics for biologists contains articles on many of the points above.

Software and code

Policy information about availability of computer code

Data collection	Fiber photometry data was collected with Bonsai v2. Place aversion assay collected with Ethovision XT v17. Motor behaviors captured with Digigait software. Place preference and EPM collected with Ethovision XT v17.
Data analysis	Analysis of Z-scored delta F/F was performed in Matlab R2023b as described in Martianova et al., 2019 and readily available at https:// github.com/ katemartian/Photometry_data_processing. Two photon tomography images processed and aligned using MBF NeuroInfo v1. Motor behaviors analyzed with Digigait software. Place preference and EPM analyzed via Ethovision XT v17. Data statistically analyzed by Graph Pad Prism v9.

For manuscripts utilizing custom algorithms or software that are central to the research but not yet described in published literature, software must be made available to editors and reviewers. We strongly encourage code deposition in a community repository (e.g. GitHub). See the Nature Portfolio guidelines for submitting code & software for further information.

Data

Policy information about availability of data

All manuscripts must include a data availability statement. This statement should provide the following information, where applicable:

- Accession codes, unique identifiers, or web links for publicly available datasets
- A description of any restrictions on data availability
- For clinical datasets or third party data, please ensure that the statement adheres to our policy

All data from this manuscript are available in the Source Data. Data from Allen Brain Atlas was collected from https://connectivity.brain-map.org/.

Research involving human participants, their data, or biological material

Policy information about studies with human participants or human data. See also policy information about sex, gender (identity/presentation), and sexual orientation and race, ethnicity and racism.

Reporting on sex and gender	N/A
Reporting on race, ethnicity, or other socially relevant groupings	N/A
Population characteristics	N/A
Recruitment	N/A
Ethics oversight	N/A

Note that full information on the approval of the study protocol must also be provided in the manuscript.

Field-specific reporting

Please select the one below that is the best fit for your research. If you are not sure, read the appropriate sections before making your selection.

☒ Life sciences ☐ Behavioural & social sciences ☐ Ecological, evolutionary & environmental sciences

For a reference copy of the document with all sections, see nature.com/documents/nr-reporting-summary-flat.pdf

Life sciences study design

All studies must disclose on these points even when the disclosure is negative.

Sample size	Sample sizes, specifically for behavior, were determined by power calculations based off of preliminary data and standard deviations. For anatomical tracing, no sample sizes were predetermined but chosen based on published literature and all results from experiments were similar. For electrophysiology, sample sizes were chosen based on published literature. For fiber photometry, sample sizes were chosen based on preliminary data and the standard variations observed.
Data exclusions	Data were only excluded when an animal needed to be medically euthanized.
Replication	All attempts at replication were successful. Specifically, The PV-mCherry/ChR2 behavioral experiments presented in Fig. 1g, i, j, n were repeated independently 3 times with 3-4 animals per group with similar results. The fiber photometry experiments in Fig. 2 were repeated independently 3 times with 1 animal per group with similar results. The anatomical tracing experiments in Fig. 3f, were performed twice independently with two-photon tomography and once with serial histology with similar results. Retrograde tracing in Fig 3g/h and 3g/i, was performed 4 independent times with 1 animal per group and 2 independent times, respectively, with similar results. Rabies tracing in Fig. 4 was conducted with 3 animals per group with similar results. The DREADD behavioral experiments in Fig. 5 d-j were repeated 4 times independently with 3-5 animals per group with similar results.
Randomization	All animals were randomly assigned to viral injection and test groups accordingly.
Blinding	Investigators were blind to all experiments.

Reporting for specific materials, systems and methods

We require information from authors about some types of materials, experimental systems and methods used in many studies. Here, indicate whether each material, system or method listed is relevant to your study. If you are not sure if a list item applies to your research, read the appropriate section before selecting a response.

Materials & experimental systems

n/a	Involved in the study
☐	☒ Antibodies
☒	☐ Eukaryotic cell lines
☒	☐ Palaeontology and archaeology
☐	☒ Animals and other organisms
☒	☐ Clinical data
☒	☐ Dual use research of concern
☒	☐ Plants

Methods

n/a	Involved in the study
☒	☐ ChIP-seq
☒	☐ Flow cytometry
☒	☐ MRI-based neuroimaging

Antibodies

Antibodies used	anti-mCherry (Abcam: ab167453), anti-Foxp1 (Abcam: ab16645 1:500), goat anti-rabbit 568 (Thermo Scientific: A-110011), goat anti-rabbit 488 (Thermo Scientific: A-11008)
Validation	mCherry antibody: Validated for use by over 300 papers including von Ziegler LM et al. Multiomic profiling of the acute stress response in the mouse hippocampus. Nat Commun 13:1824 (2022).<br>Foxpl antibody: Validated by over 90 publications including Liau ES et al. Single-cell transcriptomic analysis reveals diversity within mammalian spinal motor neurons. Nat Commun 14:46 (2023).

Animals and other research organisms

Policy information about studies involving animals; ARRIVE guidelines recommended for reporting animal research, and Sex and Gender in Research

Laboratory animals	All mice were kept in a 12 hour light/dark cycle, at 70 degrees F, with 30-50% humidity. All mice were enrolled between 10-14 weeks of age. Strains used include: C57BL6/J (Jax #000664) B6.129P2-Pvalbtm1(cre)Arbr/J (Jax #017320), Penk-Cre (Jax # 025112), Rbp4-Cre (MGI:4367067)
Wild animals	N/A
Reporting on sex	Both sexes of mice were included in the analysis throughout the paper in roughly equal numbers. This is reported in the Source Data.
Field-collected samples	N/A
Ethics oversight	Ethics oversight was provided by the Boston Children's Hospital Institutional Animal Use and Care Committee (IACUC) under protocols 00001507, 00001546, and 20-05-4165.

Note that full information on the approval of the study protocol must also be provided in the manuscript.

Plants

Seed stocks	N/A
Novel plant genotypes	N/A
Authentication	N/A
